# Consequences of top-down knowledge hiding in firms: A pilot study

**DOI:** 10.1016/j.heliyon.2019.e03000

**Published:** 2019-12-07

**Authors:** Atif Saleem Butt

**Affiliations:** Department of Management, School of Business, American University of Ras Al Khaimah, Ras Al Khaimah, United Arab Emirates

**Keywords:** Business, Knowledge hiding, Management, Pilot study

## Abstract

While knowledge hiding has been the subject of some research in the management discipline, extant literature is scant on the consequences of top-down knowledge hiding in firms. Therefore, the purpose of this paper is to explore the individual-level consequences of top-down knowledge hiding in firms. Based on an initial pilot study, the preliminary findings unveil three types of individual-level consequences of top-down knowledge such as loss of personal reputation of knowledge seeker, lack of creativity and lack of productivity. This paper discusses its initial contribution to knowledge hiding literature and concludes by discussing its limitations and scope for future research.

## 1 Introduction

Knowledge hiding has been the subject of a lot of research in the business domain. Numerous ongoing studies have contended that knowledge hiding can negatively affect people (Butt and Ahmad, 2019a, Butt and Ahmad, 2019b; Connelley et al., 2019; Cheng et al., 2008; Samuel et al., 2011; Fang, 2017). These researchers further contend that knowledge hiding can negatively affect the firms' capacity to enhance wellness and stay focused on the challenge. Moreover, Arain et al. (2018) found that intra-organization knowledge hiding can make a domain of vulnerability and it can unfavourably influence knowledge searchers basic leadership process, which can indirectly affect company's overall success. Connelly et al. (2012, p. 65) define knowledge hiding is defined as "an intentional attempt by an individual to withhold or conceal knowledge that has been requested by another person". Such knowledge hiding inside firms can hurt the company's capacity to stay aggressive and creative. Ongoing studies additionally shed some light on potential predecessors of knowledge hiding. For example, Butt and Ahmad, 2019a, Butt and Ahmad, 2019b displayed an exact model portraying outcomes of knowledge hiding up at three levels (individual, relational and firms). These authors further contended that knowledge hiders don't just conceal information in light of the individual gains yet now and then they take part in such practices to meet the standards of norms and with an intention to reciprocate. Moreover, Černe et al., (2014, 2017) expressed that people inside firms can take part in knowledge hiding in order to save their vitality and profession possibilities. Serenko and Bontis (2016) argued that it is typically knowledge hiders' narrow-minded intentions, which urge them to intentionally hide knowledge from their associates and additionally peers inside firms.

While these studies enhanced our understanding of this subject matter, studies concentrating on the potential results of top-down knowledge hiding are unknown yet. Different studies (Connelly et al., 2012; Černe et al., 2014) contend that top-down knowledge hiding affects the execution of the doled out jobs and obligations in supporting the organization to accomplish the business. For example, Butt and Ahmad, 2019a, Butt and Ahmad, 2019b contended that top-down knowledge hiding may compel victims of knowledge hiders to voluntarily resign from their position. Consequently, this pilot study endeavours to uncover potential outcomes of top-down knowledge hiding in firms. The following research question is proposed to guide this pilot study: *How does knowledge hiding between supervisor-subordinate relationship affect subordinate's work performance*?

## 2 Literature review

### 2.1 Knowledge hiding

Intra-organizational knowledge hiding can have negative results for employees. For example, Connelly and Zweig (2015) and Ann Louise Holten et al. (2016) detailed that knowledge hiding between two collaborators antagonistically influences knowledge seekers' capacity to be imaginative and creative. Labafi (2017) has additionally advised that knowledge hiding can hurt representatives' efficiency. These studies argue that there may be additional outcomes of top-down knowledge hiding. For example, victims of knowledge hiding may likewise feel their positions in question as they may not have enough information to play out their job to the best of their potential. While studies contend that knowledge hiding can antagonistically affect knowledge seekers, it merits referencing that the impacts of knowledge hiding can likewise be felt at relational and firm-level. For example, employees who become a victim of knowledge hiding may leave their positions. Such volunteer turnover can be expensive for firms (Butt, 2018; Sharma and Singh, 2014; He et al., 2009; Yeşil and Hırlak, 2013). In different occurrences, Fong and Slotta (2018) and Laframboise et al. (2007) likewise contended that knowledge hiding doesn't just affect individuals, however firms also. Specifically, they battled that knowledge hiding in firms can really bring about higher turnover among knowledge seekers. Butt and Ahmad, 2019a, Butt and Ahmad, 2019b, further argued that top-down knowledge hiding can influence the association's notoriety in the challenge, bringing about decreased business volume. knowledge hiding can likewise further influence the connection between culprit and knowledge searcher. For example, Serenko and Bontis (2016) stated that knowledge hiding between associated parties (information searcher and information hider) can influence the trust level among them, and they may not have a similar degree of bonding with regards to significant business errands. Černe et al. (2014) contended that the connection between supervisor and subordinate can prompt the doubt circle. Connelley et al., (2019), has likewise revealed that knowledge hiding among culprit and information searcher can hurt their business relationship in the domain of trust and dedication while Wang et al. (2019) contended that intra-organizational knowledge hiding can build execution in the business and fills in as a positive execution. With everything taken into account, these ongoing investigations contended the impacts of top-down knowledge hiding can rise above from information searchers to a connection between information searchers and culprits just as firms. Connelly et al. (2019) has made calls to investigate the individual-level results of top-down knowledge hiding. Consequently, a pilot study was performed to analyze the potential outcomes that knowledge seekers encounter when their senior managers intentionally hide knowledge from them in the firms.

Consequently, the researcher contacted managers through one industrial platform. The managers included in this study came from a variety of industrial and service background. The managers' view on the negative impact of knowledge hiding on knowledge seeking individual was recorded through extensive interview notes (Creswell, 2013). The researcher used some secondary data to ensure triangulation, such as the company's annual reports, website, and social media (e.g., blogs, YouTube, and online magazines). These multiple and heterogeneous data sources contribute to a converging line of inquiry (Yin, 2009). It is also important to mention that the researcher's affiliated institute did not require ethical approval for this study.

## 3 Results

### 3.1 Personal reputation

Discussion with knowledge seekers (respondents) thoroughly revealed that they felt their reputation at stake once they did not have full knowledge to perform their roles. Interviews with two respondents stated that it is very common in their organization for senior management to deliberately hide knowledge from their peers as well as subordinates. Even worse than that, respondents confirmed that there is no mechanism in their firm to report such issues to the top-level management so knowledge hiding behaviours can be mitigated. These respondents narrated that, their peers around them began to feel that respondents do not know how to do their job properly or exceed their role expectations in order to earn a good reputation. One of the interviews stated that.

*My boss deliberately kept me in the corner and did not take me on board when I asked him to assist in designing a new customer database for one of our clients. I had a good reputation for handling such projects when I entered the firm initially but now colleagues believe that it's not eventually the case. They are not aware of the reality that I am purposely kept out of this project assignment. What is even worse that nobody cares about this prevailing knowledge hiding behaviour in our organizations. There is no mechanism and system in place to cope with this issue.*

Two interviews with knowledge seekers stated that they are usually get hired based on your competencies and skills that you are going to bring into this company. However, if your peers don't see you delivering, they began to doubt your identity at a professional level. These respondents contended that they are, actually, fully motivated to work with their seniors on a one-on-one basis, but their senior managers don't take them on board, neither they cooperate fully. These knowledge seekers argued that knowledge hiding perpetrators possess a strong reputation in the company because they hold that specific knowledge and it adversely affects the knowledge seekers reputation on the other side when knowledge is deliberately hidden from them. Like respondents previous respondents, they confirmed that their companies do not have any mechanism and policies in place to take any strict action against these perpetrators.

Initial interviews also provided support to the above findings. For instance, one respondent argued that when you do not have full knowledge to perform your job, you are not going to be in good books of your boss even though it is clear that your supervisor is not cooperating well with you or supporting in terms of acquiring new knowledge. This respondent further stressed that your personal reputation is always felt at stake and sometimes employees in the competition also know that you do not have full knowledge to perform your role, which precludes you from applying for better positions in the competition. This respondent also contended that when your personal reputation is lost as a result of lack of knowledge, you are no longer respected in the firm and begin to lose your real value. Moreover, firms take no initiative to sort out what it might not really be your mistake. The respondent further argued that unfortunately, his firm does not have any policies in place which held the perpetrator accountable for failing to provide the required knowledge to the knowledge seeker. Consequently, much needs to be done to make sure that when knowledge hiding culture prevails in firms, perpetrators are identified and held accountable for their unethical behaviours rather than pointing out knowledge seekers who are not really performing their role properly due to the lack of knowledge.

Additional findings also suggest that when knowledge seekers fail to gain access to the required knowledge from their superordinates, this affects their ability to be fully functional within the confines of the organizational role. In other words, they are not able to perform their role well and begin to make mistakes due to lack of knowledge. Findings also state senior managers then begin to put blame on them and they begin to lose their dignity and respect in the firm. Furthermore, their bad reputation spans across the boundary of an organization and reaches other competitors as well. Findings suggest that when managers’ personal reputation is hurt due to the lack of knowledge they possess, this adversely affects their productivity level. In fact, findings unveil that, sometimes, their bad reputation sometimes takes them in deep strain, encouraging them to quit their jobs or move onto a different company. This is alarming for firms because if managers decide to quit from their role out of the fear that they are not being valued in a company, such turnover can be quite costly for the firms.

### 3.2 Lack of creativity

Respondents’ stories thoroughly revealed that they were not able to be innovative and creative in their roles because they did not have full knowledge to perform their jobs. Respondents also narrated that they did not know how to handle their tasks in a different way because they lacked the knowledge to do so. Furthermore, respondents blamed their senior managers for their lack of creativity because they did not provide them with the full knowledge to bring creativity in their roles.

Interviews also unveiled that their senior managers willingly neglected them when they approached them with a request to train them on a new software project. These two respondents narrated that the clients wanted their company to solve issues relating to updating customer query database but that required a new and innovative way. Respondents narrated that they were so keen to learn about that project and were willing to bring new ways of updating their customer's database query but they could not do so because their senior managers were not prepared to take them on board and said that its not the right time for them to handle this big project. The respondents further narrated that their senior managers never requested them to attend a meeting to discuss the new ways of handling the project even when they made several requests to be a part of the team.

The issue of lack of creativity was further confirmed by another interview. This respondent thoroughly argued that when you do not have access to the full knowledge, this is going to adversely affect your ability to be creative. For example, you might not be able to come up with new and innovative ideas that might help you win new projects from your customers. In other instances, sometimes even you are sincerely willing to develop a new product or improve the design of the current product, you cannot do so as your top management does not train you or give access to the knowledge that you need to be creative. This respondent argued that creativity is an absolutely essential element in firms success. If managers are not creative, firms cannot compete well in the competition, but, unfortunately, firms do not realize how intra-organizational knowledge is actually damaging the creativity of the employees. Consider the following excerpt.

*You need complete knowledge to be creative, this is as simple as it is. We have people around who do not share knowledge with their junior for several reasons. As a result, they cannot come up with new ideas to help the company innovate, which affect our relationship with customers.*

Furthermore, findings also unveil that lack of creativity among managers as a result of knowledge hiding adversely affect the everyday business of the firms. Firms are not in a position to profit and consequently, would not be accepting new ideas. In addition, when managers lack creativity as a result of knowledge hiding, this will decrease the satisfaction level among employees in the firms. Furthermore, preliminary findings also suggest that when knowledge hiding results in reduced creativity among employees of the firms, this eventually result in lower customer satisfaction in the present and future. Furthermore, it does not matter how technologically advanced a company is, employees who are less creative will be less satisfied with the companies they work for.

In addition, as knowledge hiding pose a serious threat to reduced creativity among employees in firms, results argue that supplying firms may not be able to make a product that customer is going to admire and managers who are the victim of knowledge hiding do not have an opportunity to stand out in the competition and industry. Furthermore, findings do suggest that when employees do not have full knowledge to perform their role, they begin to model their competition or industry, which slows down their business or even the firm is left in isolation and dies in the minds of their customers. All in all, firms lack the opportunity to be different from their customers.

### 3.3 Lack of productivity

Findings also suggest that when senior managers intentionally hide knowledge from their subordinates, this affects their level of productivity as well. Findings suggest employees who do not have full knowledge to perform their roles are no longer deliver the highest outputs possible and struggle with their roles. Furthermore, there is a direct correlation between knowledge hiding and managers' reduced level of productivity, which ultimately impacts the profits of the company. Findings also state that when managers lack productivity as a result of knowledge hiding, this can result in employees termination in the firm and managers subsequently begin to suffer from the low morale based on poor working relationship with the perpetrator. Firms, on the other hand, do not take an initiative to set an agreement between knowledge seekers and perpetrators to make sure that employees remain productive. Findings also state that managers who lack the knowledge to perform their roles are unable to set or achieve business goals, or they may not be in a position to manage their priorities effectively. This can, on the whole, lower the employee's morale and build resentment.

Notably, findings contend that knowledge hiding affects the productivity of the victims of knowledge hiding, which in turn results in decreased motivation. This also impacts firms, as they begin to experience high absenteeism among employees and turnover. Knowledge hiding compels managers to remain, low producing workers, calling in sick frequently based on their limited value in the company. Turnovers begin to increase as employees are no longer motivated because they do not have full knowledge to perform their role and do not feel that their contributions are valued as well.

Most importantly, when managers are the victim of knowledge hiding, the chances that they will do well in their daily tasks begin to diminish including reduced sales, lack of customer satisfaction and tasks may take much more time to be completed. Furthermore, they might not be in a position to fill orders in a time manger, or otherwise, perform their job functions and stay on task run, letting firms lose the business. This adversely affects the customer's confidence in suppliers and customers may eventually take their business elsewhere. So, when managers do not have full knowledge to perform their role, it decreased the competitive edge that a business has which can have a serious financial effect on the bottom line, leading to the closure of the business. Victims of knowledge hiding are not in a position their jobs properly. Furthermore, preliminary findings also suggest that managers who are victim of knowledge hiding are become less productive, which in turns increase stress level among them, particularly for employees, who really value their role and company. This creates tension and a sense of hopelessness among employees because they are unproductive due to the culture of knowledge hiding in firms. Such employees can potentially leave jobs and switch to new employment. Finally, managers who are a victim of knowledge hiding can see their long-term performance affected badly and they may not be able to meet project deadlines or may not be able to complete their goals within a reasonable amount of time. All in all, a lack of knowledge means that there are fairly high chances that your competition is going to supersede and take advantage of you.

The issue related to loss of productivity continued to emerge from this pilot study. The initial findings suggest that knowledge hiding had an outright impact on a manager's productivity and adversely affect their efficiency level. For instance, when knowledge seekers lack the knowledge to perform their role, it adversely affects their long and short term goals, their ability to prioritize tasks, notably in a small and medium-sized business (SMEs). Furthermore, victims of knowledge hiding do not have a clear and strong sense of work ethics and they are low performing too. Perhaps, providing an incentive when they share knowledge within organizations can improve their productivity level and help them set goals which are both realistic and achievable. Initial findings also argue that a lack of knowledge means managers are no longer encouraged, motivated and rewarded and their peers or senior never encourage them they are doing something great.

## 4 Discussion

Our preliminary findings argue that when senior management intentionally hide knowledge from their subordinates, this is going to negatively impact the victim's reputation. In fact, knowledge seekers are left isolated and have no value in the competition. Furthermore, initial findings further argue that top-down knowledge hiding also affects managers creativity and supplying firms struggle when it comes to building or retaining strong clients. Butt and Ahmad, 2019a, Butt and Ahmad, 2019b also argued that when managers from the supplying firms do not have full knowledge to perform their job, they will find it challenging to maintain a good relationship with their customer base. In fact, they will begin to lose clients. Moving on, initial results also unveil that knowledge hiding negatively impact managers' ability to contribute to the best of their potential and to produce the required amount of outputs. This further provides empirical support to the studies on knowledge hiding arguing that knowledge hiding between two co-workers can affect knowledge seekers' ability to be creative as well as innovative (Connelly and Zweig, 2015; Ann Louise Holten et al. 2016).

Findings from our study can provide an initial idea to firms on how top-down knowledge hiding in firms can have a negative impact on knowledge seekers ability resulting in higher stress level and declined motivation. Furthermore, such a higher stress level and declined motivation can compel victims of knowledge seekers to voluntarily resign from their positions. Firms should, therefore, note that such turnover could be costly for firms and it can result in huge productivity loss. Firms should also note that while employees can certainly be replaced, time is needed to adjust in a new culture. Perhaps, the development of friendly ties between the supervisor and their subordinates could be paramount as this can help cultivate trust and sense of belongingness. Recent research Butt, 2019a, Butt, 2019b, Butt, 2019c, Butt, 2019d, Butt, 2019e; Butt and Ahmad, 2019a, Butt and Ahmad, 2019b; Butt et al. (2019); Ahmad et al. (2019) have also argued that development of friendly ties between managers within a different hierarchical level can mitigate knowledge hiding in firms. Moreover, induction and socialization process can be time-consuming and such efforts could be spent on enhancing the firm's competitiveness in the market perhaps. It is also important to mention that knowledge seeker can potentially decrease (or perhaps do not have any negative impact on them) as a result of knowledge hiding. However, this is only going to be possible, when managers are capable enough to take an initiative and approach the perpetrators who are deliberately hiding knowledge from them to discuss the prevailing issue. Serenko and Bontis (2016) have also suggested that knowledge hiding may not necessarily always generate negative consequences. However, this requires managers to escalate any conflicts and issues matter with their respective superordinates outrightly.

## 5 Conclusion

While the findings from this pilot study tell us about the consequences of top-down knowledge hiding, it is pertinent to mention that it is a pilot study, which attempts to reveal managers’ initial perception of the possible individual-level consequences of top-down knowledge hiding. Furthermore, the research question of this study specifically addresses behavioural patterns in United Arab Emirates Culture only. Also, this study relies on cross-section data to explore behavioural patterns on these phenomena, which limits the extent to which cause-effect relations can be inferred.

There are further avenues for future research. For example, respondents contended that there could be numerous extra outcomes of top-down knowledge hiding at individual-level. Hence, future research should dive further into this subject by gathering information from a bigger populace size as this will fortify and improve our comprehension of this point. In addition, preliminary data recommend that top-down knowledge hiding can't just influence the knowledge searchers, yet it can likewise negatively affect groups as well as firms. It would, consequently, be fascinating to explore whether the effects of top-down knowledge hiding range over various levels (people and groups and so on.) and whether such outcomes could be multifaceted as well. Another extension of this topic is to gather dyadic information from the subordinates and their ranking super-ordinates to affirm the discoveries as subordinates may not really have the full picture, and there could be different predispositions emerging from individual connections or misguided judgments. Moreover, future research ought to examine how knowledge hiding can be relieved in firms as information yield that a few firms have shown a drive to alleviate the way of life of knowledge hiding by settling on specific techniques and policies. Besides, investigating how knowledge hiding can be relieved in firms can assist firms with beating the negative results related with. At last, future research should investigate how companies can create strategies that can empower the culture of knowledge sharing while simultaneously disheartening the way of knowledge hiding in firms.

## Declarations

### Author contribution statement

A. S. Butt: Conceived and designed the experiments; Performed the experiments; Analyzed and interpreted the data; Contributed reagents, materials, analysis tools or data; Wrote the paper.

### Funding statement

This research did not receive any specific grant from funding agencies in the public, commercial, or not-for-profit sectors.

### Competing interest statement

The authors declare no conflict of interest.

### Additional information

No additional information is available for this paper.
